# Proceedings of the New York Homœopathic Union

**Published:** 1894-03

**Authors:** 


### PROCEEDINGS OF THE NEW YORK HOMŒOPATHIC UNION.

The regular monthly meeting of the New York Homœopathic Union was held at 53 W. Forty-fifth Street, New York, December 21st, 1893, the President, Dr. Edmund Carleton, in the chair. There were present Drs. Givens, Finch, Woodruff, Harman, Fincke, Campbell, Alice B. Campbell, Clock, Dyer, Thomson, Winterburn, Davis, Powel, and O'Connor. Letters of regret were read from Drs. S. H. Talcott, C. S. Kinney, W. M. James, and F. C. Donovan.

The subject of discussion was §§ 210 to 230, inclusive, of Hahnemann's *Organon*, relating to Mental Diseases.

Dr. Fincke—I see that, in the first paragraph of Stratten's translation, it is given "*diseases of the mind and temper.*" I would like to have members express themselves as to the meaning of these words. I note that Dudgeon has it "*mental and emotional diseases.*" I think that is about as well as it could be expressed. I don't know what the general opinion is, but it seems to me that the *spirit itself* could never get diseased.

Dr. Dyer—I think if Hahnemann intended that it should be "*mental diseases*," that's the way it should be translated.

Dr. O'Connor—Hahnemann has it all right, but there is a little difference in the meaning in the German, which makes some difference in the rendering. If I were translating it, I should insist upon using both terms. First, there is simply the disturbance of what is meant by the whole activity of our mentality from the affected side; next, there will be the disturbance of the intellectual side and the emotional, or just the emotional; and the third division, psychical degeneration—*i. e.*, coming from the parents—idiocy—imbecility—the so-called moral insanity, which is really a moral weakness. Those three divisions are the three classes.

Dr. Finch—Is not this psychical degeneration a degeneration in his starting—in the soul, in the mind?

Dr. O'Connor—That brings up another question. We use it in the sense of our psychical activity with mental disturbances of the soul.

Dr. Fincke—Hahnemann makes this distinction, and I think it's a very good one.

Dr. Thomson—Is it a fact that Dudgeon has it as Hahnemann intended it? Wesselhœft has it "*mind and temperament.*"

Dr. O'Connor—He has got his own temperament. I am speaking of a change in the man's make-up. You can't change that by any medicine.

Dr. Dyer—Suppose he is born sick?

Dr. O'Connor—If he can't be relieved, he has got to go through life with it. You might as well talk of changing the shape of a man's nose.

Dr. Dyer—The character changes the shape of a man's nose. When they are young—

Dr. Thomson (interrupting)—That's a different thing. We are changing our forms according to the way we are acting—good or ill, according to the principles which actuate us; and the temperament isn't changed.

Dr. O'Connor—I said you can't change it by treatment with medicine.

Dr. Dyer—If the disposition can change the features then you can change it by medicine.

Dr. Fincke—A case comes to my mind which shows that sometimes a change may take place. A girl I knew had Ether administered to her; and after the etherization she turned quite the other way. It changed her whole disposition and she remained so. This matter of temperament is a thing that would naturally come under consideration, for in these diseases temperament must be considered.

Dr. Thomson—If selecting a remedy for these diseases is to be considered, I should like to hear from the gentlemen who make a specialty of nervous diseases.

The President—There are three of these physicians present, Drs. Givens, O'Connor, and Davis.

Dr. O'Connor—Hahnemann mentions mental emotional diseases that spring from the body, and then those that are indigenous, so to say, that spring from themselves. I agree entirely with what is said in paragraph 224.

Section 225 was then read by Dr. Finch.

Dr. Fincke—Some will take their action from the body and some from the mind.

Dr. Dyer—I would like to ask Dr. O'Connor a question. Perhaps I misunderstood him. Am I right in understanding you to say that you cannot change a person's temperament without creating disease?

Dr. O'Connor—What I said was that it is impossible to change the actual temperament by any medicine. Hahnemann shows the changes in disposition in the changes that take place in that sense, where he shows that the man who is mild and still, as soon as he becomes sick becomes profane and violent, and one who is cheerful and nice will become low spirited and melancholy. He offers this as a proof that the individual is made sick.

Dr. Dyer—I don't think you have answered my question.

Dr. O'Connor—I agree with Dr. Thomson that you cannot change the actual disposition of the patient. The patient under the influence of some great mental emotion or drug becomes sick and—

Dr. Dyer—Is his temperament then changed?

Dr. O'Connor—For that time. The individual is not going to be changed and be healthy.

Dr. Dyer—That's what I mean. You mean that he cannot have his temperament changed and remain in a healthy state; but that, by drug action or by shocks, he may be changed temporarily? Well, that's my point.

Dr. Finch—After all, it will depend on what definition you give the word temperament.

Dr. Thomson—It is suspension of temperament. It's like the old song: "I'm not myself at all, Mollie dear." His temperament is suspended, like all the other things.

Dr. Fincke—I think we are drifting too much into psychology. There is the sanguine, the choleric, the lymphatic, and the nervous temperament. Now, if you please, they never occur in any person exclusively. There is always something else mixed in it; so I say let us all go to Hahnemann and take up the features of the disease and the changes in the mind, and go according to those symptoms, and we will be right.

Dr. Finch—I may mention right here a little experience with a drug as related to temperament. Take the light hair—the sandy hair and the red hair—and a peculiar conformation of features denoting the sanguine-lymphatic; take that mixture, and comparing with what were termed the bilious, dark person, and treat them with Podophyllum, as I did a great many years ago, from the third attenuation down to the crude substance; and to the bilious temperament—the dark complexion—you might give a grain of the Podophylline, the alkaloid—and they would go to sleep on it and never think of it again; while to the sanguine, mixed with the lymphatic, individual it would give a chronic diarrhœa which I have known to last three weeks.

Dr. Woodruff—Have you known that to occur often?

Dr. Finch—It has occurred many and many a time with me and with friends of mine. You will find it true of Mandrake that it has a much greater influence upon the temperaments I have named, the lymphatic and sanguine, than upon the others which I have also mentioned. Given alone and on a dark-complexioned individual, I have known it to nauseate, but to produce no discharges whatever; and on a light-complexioned person, like on our worthy President, it would produce an effect lasting for a long time. It is only a little matter of experience, showing that certain drugs affect certain temperaments more than they do others.

Dr. Thomson—A certain physician claimed that by looking into the face he could judge of the disease the person would be subject to; upon which, another physician present at the time, who had light hair and lymphatic temperament, said, "Well, what do you say to one of my temperament? What disease should I be subject to?" He had never seen the man before, but he got upon his feet and said, "I am an Englishman, and never am challenged without replying. Hemorrhage! sir; hemorrhage! from the day of your birth to the day you die." The other replied, "It is what I have always feared."

Dr. Harman—I know that disposition to hemorrhage is true. I am now treating the case of a little boy who is subject to hemorrhages monthly. He is born of a hemorrhagic mother and bleeds everywhere. It comes from the nose and at times from the eyes. I have seen a little blister rise in a few moments on the tongue and bleed.

Dr. Fincke—There was an old French physician in Lyons, Dr. Gallavardin, whose experience you will have read in the *Medical Advance.* He made people like a certain profession. For instance, one did not like the law, and by giving him a certain remedy he changed his mind. Or some people wouldn't marry and he gave *them* a remedy, and so they were married. I suppose that would come in here as changing the mind.

Dr. Thomson—In view of the terrible torture of the insane at the time of Hahnemann, it is wonderful the higher stand that he took. He stood almost alone, and he said that the insane should be treated as being innocent as children, as irresponsible beings, and that torture or punishment of any kind or description, no matter what they did, was an evil and a wrong to humanity. It makes Hahnemann stand personally, philosophically, and religiously above others of that day and even of our present day, except a few who are following in his footsteps.

Paragraph 229: "*The physician, and those who guard the patient, ought always to appear as if they believed him to be possessed of reason.*"

Dr. O'Connor—Except I do not think they ought to make truth of his hallucinations. To avoid denying their sanity I think is a part of wisdom. I think it is very bad to make believe you believe in what they have said. Simply be quiet or try to account for it in some way.

The President—I saw a fine piece of management of a case where a patient insisted upon it that a cloud was a camel. Dr. Bayard said: "You call that a camel? I call it a cloud; but we won't quarrel about that." He followed just that calm way of reasoning all the time. If patients have to go to an asylum, they should not be deceived about it.

Dr. O'Connor—I don't believe I agree with you there. Sometimes we have to resort to some kind of deception. Hahnemann says that some little deception must be used. He did not believe in deception if it could be avoided.

The President—In giving them medicine I believe in it; but I have tried both ways in getting them to an asylum, and I believe the best way is not to deceive them. Just say to them: "Now, you have to go. If you are determined to make a fuss, and make trouble for yourself and friends, you have to go, just the same." What do you think, Dr. Givens?

Dr. Givens—My experience is that it is best to get the patient to the *hospital* without any deception. The deception may be a little easier for the friends at the time but it is always worse for the patients. They do not forget those things immediately.

Dr. Davis—In exceptional cases you have got some time to do it.

Dr. Givens—I recall a case that came to Middletown. He came in charge of four policemen and weighted down with chains. He was reported as a very dangerous character. We asked why they brought him there in that shape, and told them to take everything off. After we took them off, he behaved all right. He thought it wrong the way they brought him there.

The President—Now, query, is it justifiable deception when you find that you can't convince them of their error, to fall in with it? I think this is not only permissible, but sometimes commendable. I am thinking at the present moment of two cases in point. The first is mentioned by Gross. A gentleman became possessed of the notion that a big tumor was on the end of his nose. Every physician whom he consulted, tried to dispel his illusion; but that only made him furious. At length a shrewd medical man in England who had heard of all this, learned that the monomaniac proposed to consult him. The meeting took place. The physician appeared to be amazed at the big tumor, but said he would at once remove it. He incised the skin slightly, at the tip of the nose, and held up in triumph the supposed tumor, which was a ham he had had concealed. The man was cured.

Case number two came to my notice, after the subject of it had exhausted the patience and skillful prescribing of other physicians. A well educated gentleman, of social and professional standing, conceived the idea that his nose was too large and ugly. Cuts 1 and 2 show you that this notion was all wrong. Nothing could shake his determination to have his nose reduced in size and made as nearly Roman in shape as possible. After a long siege of reasoning and prescribing, I yielded to his importunities, incised the skin, exposed the parts beneath, trimmed them to the required outline, made the skin correspond, took numerous, fine stitches and accomplished the desired result. See cuts 3 and 4. His monomania was cured, and his appearance was really improved also.

Dr. O'Connor—That was a case of hypnotism. Anything that would satisfy the man, for the man would be relieved and made all right. That man isn't well; but his trouble has not gone down deep enough to have seized upon the structure of his brain, and the encouragement he gets from temporary relief is sufficient to stimulate him, and until some other condition comes upon him, some fit of depression, or a row with somebody—

but you let one of those come, and his hallucination will return —not necessarily the same delusion.

Dr. Fincke—If any one had told him they were practicing the deception upon him, he would have had the big nose again.

Dr. Thomson—Hence the man wanted speaking to in the right way and the right homœopathic remedies; and from the circumstances, I should judge that he will still require the remedy.

Dr. Dyer—That's the point I wanted to make.

Dr. Thomson—Here are two men starting in life. One by some hallucination is made to suffer all through life. It seems to me that even if he lives, he doesn't go through life with that full force of his whole organism and with the same even balance that the other does. A remedy must be found which can put the man upon a mental plane better than anything else can possibly do.

Dr. O'Connor—I think it is very hard to find the remedy. I get away from them if I can. Send them to Givens. In certain conditions of degeneracy or psychical affection—that is, degeneration from the parents—moral insanity some call it, I think hynotism has done wonders. I went to Nancy especially to study it, and I saw a great deal in Paris, but I came to the conclusion that there was so much fraud in it right there under Professor Louis that I never could make up my mind whether he knew he was a fraud or whether he was self-deceived. After a few trials here, I felt that it was better for me not to touch it, and I can't afford, for the little good that might be done, to run the risk of being classed with the mountebanks that go about. There are a few that can. They are the hysterical, weak, and nervous, and sometimes we are forced to use hypnotism; but there is a good deal in what Sharpell said, "You are setting up another disease." I don't believe in it.

Dr. Thomson—I'm glad to hear you say that. I don't believe that any human being ought to be subjected to the will of another under any circumstances or pretense whatever.

Dr. O'Connor—I would like to just state this. It isn't necessary that the individual should be subjected to my will in order to get the benefit of what is called hypnotism. I will give you a case in point: Some three years ago I was called to see a case of catalepsy, the form of insanity designated as catalonia. That is where it ought to be placed. That girl was in the cataleptic state. She had not eaten for I don't know how long. The food they put in her mouth would stay there for hours, and in a sort of way they managed to keep life in her. She was lying there with the waxy flexibility (?) peculiar to the disease. She wasn't very much wasted. After seeing the case I called the mother aside (I went in there in a brusque way, intensifying my own manner), and I said, in a voice I intended her to hear, " We will get her out of this. You give her this medicine and in an hour she will get up." I don't know what the medicine was I gave. I do not attribute the result to the medicine. But in an hour she did get up and she did eat. I believe that I did go through some tomfoolery. That girl got well to all intents and purposes. But she wasn't well, and I warned her mother not to let her go to church. I said, " You keep her away from sermons." She said she was very glad to hear that advice. The girl had religious mania, but she was twenty-four—five or six and couldn't be controlled, and she got back in the course of seven or eight months. I was away and when I came home they sent for me, and all that influence was of no avail the second time, and the girl went to Middletown and was up there for two or three years. They said that she was back and well again, and that she was coming to see me. Now what I had done gave that girl seven months of relief.

The hour being late the meeting adjourned.

